# K1 gene transformation activities in AIDS-related and classic type Kaposi’s sarcoma: Correlation with clinical presentation

**DOI:** 10.1038/s41598-019-42763-0

**Published:** 2019-04-23

**Authors:** Ayumi Tamanaha-Nakasone, Karina Uehara, Yasuka Tanabe, Haruna Ishikawa, Natsuko Yamakawa, Zensei Toyoda, Kiyoto Kurima, Shinichiro Kina, Masayuki Tsuneki, Yuko Okubo, Sayaka Yamaguchi, Daisuke Utsumi, Kenzo Takahashi, Hirofumi Arakawa, Akira Arasaki, Takao Kinjo

**Affiliations:** 1 Division of Morphological Pathology, Department of Basic Laboratory Sciences, School of Health Sciences, University of the Ryukyus, 207 Uehara, Nishihara, Okinawa, 903-0215 Japan; 2 Department of Oral and Maxillofacial Functional Rehabilitation, Graduate School of Medicine, University of the Ryukyus, 207 Uehara, Nishihara, Okinawa, 903-0215 Japan; 3 Department of Molecular Pharmacology and Oncology, Gunma University Graduate School of Medicine, 3-39-22 Showa, Maebashi, Gunma, 371-8511 Japan; 4 Division of Oral Pathology, Department of Tissue Regeneration and Reconstruction, Niigata University Graduate School of Medical and Dental Sciences, 2-5274 Gakkomachidori, Chuo, Niigata, Niigata, 951-8514 Japan; 5 Department of Dermatology, Graduate School of Medicine, University of the Ryukyus, 207 Uehara, Nishihara, Okinawa, 903-0215 Japan; 6 Division of Cancer Biology, National Cancer Center Research Institute, 5-1-1 Tsukiji, Chuo, Tokyo 104-0045 Japan

**Keywords:** Cancer models, Sarcoma, Sarcoma, Sarcoma, Tumour virus infections

## Abstract

Kaposi’s sarcoma-associated herpesvirus (KSHV) causes both AIDS-related Kaposi’s sarcoma (KS) and classic KS, but their clinical presentations are different, and respective mechanisms remain to be elucidated. The KSHV K1 gene is reportedly involved in tumorigenesis through the immunoreceptor tyrosine-based activation motif (ITAM). Since we found the sequence variations in the K1 gene of KSHV isolated from AIDS-related KS and classic KS, we hypothesized that the transformation activity of the K1 gene contributes to the different clinical presentations. To evaluate our hypothesis, we compared the transformation activities of the K1 gene between AIDS-related KS and classic KS. We also analyzed ITAM activities and the downstream AKT and NF-κB. We found that the transformation activity of AIDS-related K1 was greater than that of classic K1, and that AIDS-related K1 induced higher ITAM activity than classic K1, causing more potent Akt and NF-κB activities. K1 downregulation by siRNA in AIDS-related K1 expressing cells induced a loss of transformation properties and decreased both Akt and NF-κB activities, suggesting a correlation between the transformation activity of K1 and ITAM signaling. Our study indicates that the increased transformation activity of AIDS-related K1 is associated with its clinical aggressiveness, whereas the weak transformation activity of classic type K1 is associated with a mild clinical presentation and spontaneous regression. The mechanism of spontaneous regression of classic KS may provide new therapeutic strategy to cancer.

## Introduction

Kaposi’s sarcoma (KS) is an intermediate malignant soft tissue tumor that primarily affects cutaneous tissue; however, it may spread to the lymph nodes and visceral organs. KS is subdivided into four types based on clinical presentation, namely, AIDS-related KS, classic KS, endemic KS, and iatrogenic KS. AIDS-related KS occurs in severely immunocompromised AIDS patients as an opportunistic infection of the AIDS-related syndrome^1,2^. AIDS-related KS develops multiple cutaneous lesions that rapidly spread, progressing as visceral lesions of the lung, liver, gastrointestinal tract, and adrenal glands, which is characterized by its aggressive presentation. Classic KS is endemic in Eastern Europe, the Mediterranean, the equatorial region of Africa^1,2^, and Okinawa, subtropical southwest islands of Japan^3^. Classic KS typically affects elderly people, with males having a greater risk. Classic KS occurs as multiple cutaneous lesions in the extremities, but the lesions are usually limited to the skin and rarely involve visceral organs. The skin lesions of classic KS are slow growing and only slowly increasing in number, with some lesions showing spontaneous regression^1,2^. Although partial regression of AIDS-related KS by imatinib or antiretroviral therapy with chemotherapy were reported^4,5^, the mechanism underlying the spontaneous regression of classic KS remains unknown. Endemic African KS occurs in children and young adults in equatorial Africa, and is not related to HIV infection. The lesions of endemic KS arise as cutaneous lesions and can affect the lymph nodes. Iatrogenic KS is associated with solid organ transplantation or immunosuppressive therapy and affects the skin as well as visceral organs, with varying clinical courses^1,2^.

In 1994, Chang *et al*. isolated Kaposi’s sarcoma-associated herpesvirus (KSHV) from KS lesions^6^. Currently, KSHV is considered as a causative agent for all types of KS. Because some KSHV genes show sequence polymorphism, molecular epidemiological studies have established the genotypes of KSHV^7–9^. Among the sequence variable regions of KSHV, ORF K1, located on the 5′ end of the genome is highly variable and is utilized for genotyping of KSHV^7,8^. The K1 gene encodes a transmembrane protein with 289 amino acids, consisting highly variable, glycosylated, N-terminal ectodomain, conservative transmembrane region, and cytoplasmic domain containing an immunoreceptor tyrosine-based activation motif (ITAM)^8,10^. Through ITAM signaling, K1 protein constitutively activates the downstream Akt and NF-κB pathways, which are involved in the tumorigenesis of KSHV^11–13^. K1 has shown transformation activity both *in vivo* and *in vitro* and is regarded as an important gene associated with the tumorigenesis of KSHV^14–16^.

In a previous study, we compared the K1 gene sequence between AIDS-related KS and classic KS in Okinawa, where a considerable number of classic KS cases have been reported^17^. The K1 amino acid sequence of classic KS in Okinawa showed a five amino acid deletion in variable region 2 (VR2), and many amino acid substitutions in both VR1 and VR2 when compared with those of AIDS-related KS^17^. These results implicate that the transformation activity of K1 between AIDS-related KS and classic KS is different, and the difference may be associated with clinical presentation.

To compare the transformation activity of K1 between AIDS-related KS and classic KS, we introduced the K1 gene from these KSHV into primary mouse embryonic fibroblasts (MEFs) and compared their transformation activities. This is the first report comparing the transformation activity of the K1 gene between AIDS-related KS and classic KS.

## Results

### AIDS-related K1 induces increased cellular proliferation, whereas classic K1 shows little effect

**Figure 1:**
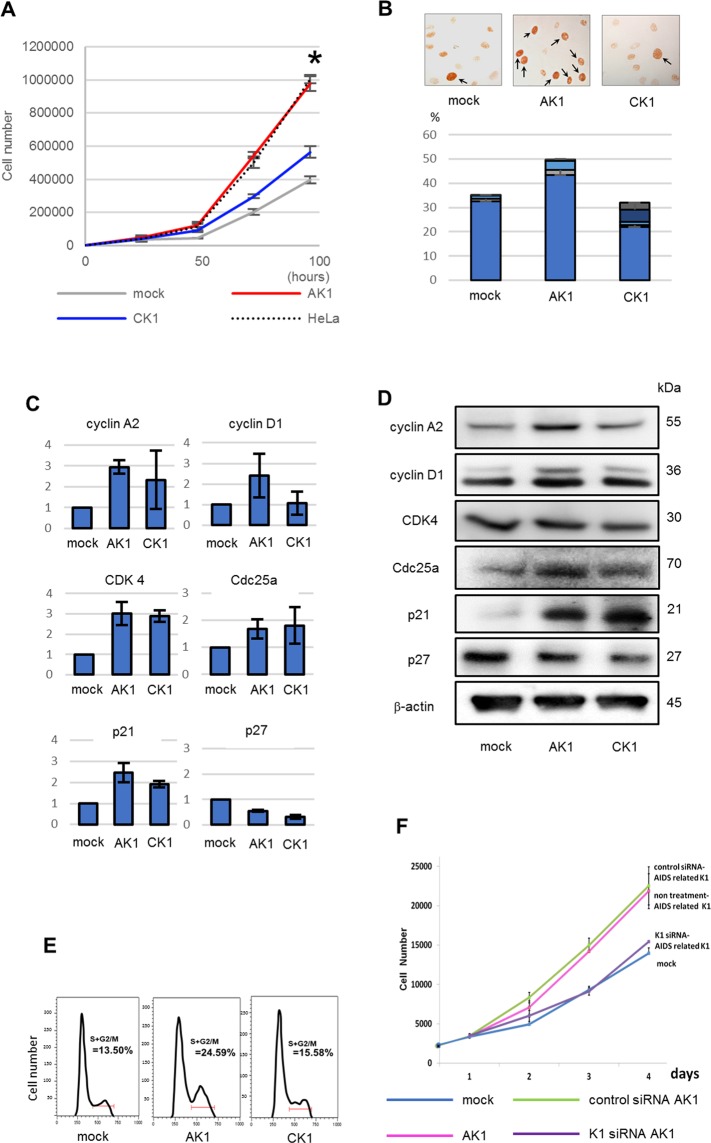
The effect of K1 expression on cellular proliferation. (**A**) Cellular proliferation assays demonstrated increased proliferation of AK1 cells. The proliferation of AK1 was comparable to that of HeLa and showed higher than that of mock with statistically significant difference *p < 0.01. (**B**) PCNA immunostaining exhibited many PCNA signals in AK1 cells. Magnification, 400×. (**C**) AK1 cells expressed high levels of cyclin A2, cyclin D1 and CDK4. Both AK1 and CK1 demonstrated much increased p21 and decreased p27. (**D**) AK1 cells showed increased levels of cyclin A2 and cyclin D1, whereas that of CDK4 was comparable among clones. In the protein levels, both AK1 and CK1 also demonstrated much increased p21 and decreased p27. (**E**) Cell cycle analysis revealed a higher population of cells in S/G2/M phases in AK1 cells compared with CK1 or mock cells. (**F**) K1 gene knockdown in AK1 cells resulted in decreased proliferation activity.

Primary mouse embryonic fibroblasts (MEFs), CF-1 line, were infected with AIDS-related K1 or classic K1 gene, AK1 and CK1, respectively, and transformation activity of K1 was assessed by comparing cellular proliferation between AK1 and CK1 cells. AK1 cells demonstrated higher rates of cellular proliferation compared with mock cells, whereas CK1 cells showed slightly higher proliferation rates to mock cells (Fig. 1A). When the S-phase marker PCNA expression were monitored, PCNA expression in AK1 cells was significantly increased compared with CK1 and mock cells, indicating an increase in the number of cells in the S-phase in AK1 cells (Fig. 1B). Real-time PCR analysis demonstrated that the expressions of cyclin A2, cyclin D1 and cyclin-dependent kinase (CDK) 4 in AK1 cells were the highest. Accordingly, cyclin A2, cyclin D1 and CDK4 expressions in CK1 cells were increased compared with mock cells (Fig. 1C). Western blot analysis also showed higher expression levels of cyclin A, cyclin D1 and Cdc25a of AK1 than those of CK1. However, protein level of CDK4 was comparable between them (Fig. 1D). Because protein levels of cyclin oscillate between synthesis and degradation in each cell cycle division^18^, as opposed to that of CDK, the results between real time PCR and Western blot showed slight discrepancy. Both AK1 and CK1 demonstrated increased expression of p21 and decreased p27 compared with mock (Fig. 1D). Since increased p21 and decreased p27 expressions are frequently observed in human cancers^18,19^, we interpreted these results as transformation caused by K1 expression. Cell cycle analysis revealed a higher population of cells in S/G2/M phases in AK1 cells than in CK1 or mock cells (Fig. 1E). Taken together, these results showed that the proliferation activity of AIDS-related K1 is higher than that of classic K1. To assess the requirement for K1 expression for cellular proliferation, we treated AK1 cells with siRNAs to knockdown the K1 gene. Knockdown of the K1 gene in AK1 cells reduced the cellular proliferation rates to levels comparable with mock cells (Fig. 1F).

### AIDS-related K1 resists apoptosis under genotoxic stress, whereas classic K1 shows only modest resistance

**Figure 2:**
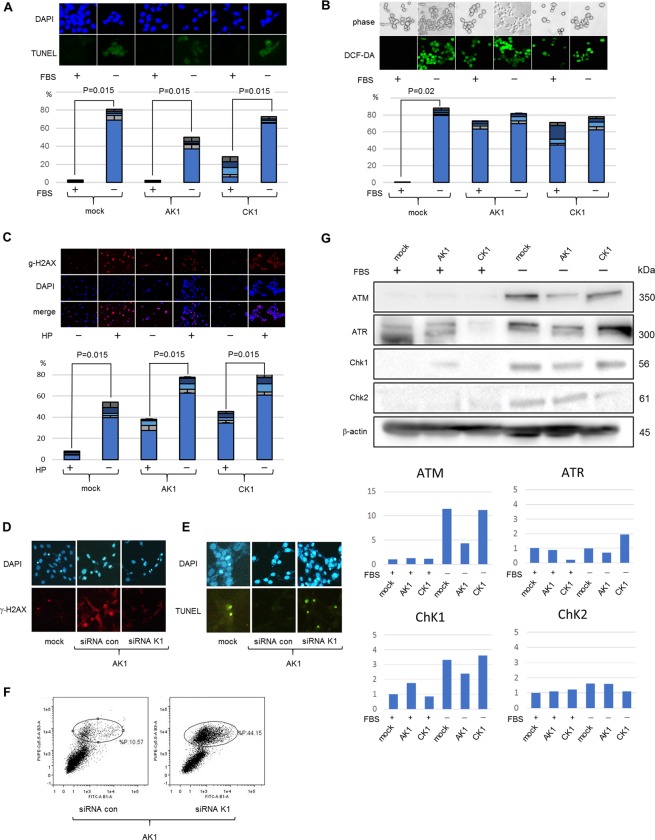
The effect of K1 expression on resistance to apoptosis, ROS generation, DNA damage and DNA damage response. (**A**) Under genotoxic conditions, mock cells showed a significant increase in the number of apoptotic cells, whereas AK1 cells demonstrated a smaller number of apoptotic cells. CK1 cells exhibited intermediate resistance. Magnification, 400×. (**B**) Under serum-free treatment, all cell lines showed increased ROS levels, with no differences among them. However, under non-genotoxic conditions, mock cells showed little ROS generation, whereas both AK1 and CK1 cells demonstrated slight increased levels of ROS generation compared with those under genotoxic conditions. Magnification, 400×. (**C**) An eightfold increase in DNA damage was observed in mock cells under genotoxic stress; however, AK1 and CK1 cells demonstrated a moderate induction of DNA damage (within twofold). Magnification, 400× *p < 0.05. (**D**) Although siRNA con-treated AK1 cells showed DNA damage, K1 knockdown demonstrated very little DNA damage. Magnification, 400×. (**E**,**F**) Under genotoxic stress, increased apoptotic cells in K1 knocked down AK1 were demonstrated by both TUNEL (**E**) and flowcytometry (**F**) analyses. (**E**) Magnification, 400×. (**G**) Under genotoxic conditions, AK1 cells strongly suppressed the induction of DDR proteins, including ATM, ATR, and Chk1, whereas CK1 cells showed only mild suppression of ATM and Chk2.

Next, we investigated cellular resistance to apoptosis associated with AIDS-related K1 or classic K1. Transformed cells are resistant to apoptosis under serum-free conditions which induces DNA damage through the intracellular generation of ROS^20,21^. When tested, the serum-free treatment provoked an increased number of apoptotic cells in both AK1 and CK1 cell lines, the degree of apoptosis in AK1 cells was considerably less than that of mock cells and that of CK1 cells was intermediate (Fig. 2A). All cell lines showed increased ROS levels, with no differences among them (Fig. 2B). To more precisely evaluate oxidative stress in these cell lines, we then compared ROS levels under non-genotoxic conditions. Although mock cells had little ROS generation, both AK1 and CK1 cells showed significantly increased levels of ROS, despite the non-genotoxic conditions (Fig. 2B). We considered that these findings stemmed from the viral gene expression in these transformed cells^22,23^. In general, increased ROS generation induces DNA damage, which triggers apoptosis under genotoxic stress, thus, we compared the degree of DNA damage in AK1 and CK1 cells. For this end, we used γ-H2AX, a DNA damage-induced phosphorylated histone that recruits DNA repair proteins, and compared the degree of γ-H2AX induction in each cell line with or without genotoxic stress. An eight-fold increase in DNA damage was observed in mock cells under genotoxic stress, whereas, both AK1 and CK1 cells demonstrated a moderate induction of DNA damage (within two-fold). The degree of DNA damage was not significantly different between AK1 and CK1 cells (Fig. 2C). These data indicate that AIDS-related K1 has an anti-apoptotic effect despite increased ROS generation and DNA damage, whereas classic K1 has mild resistance to apoptosis under genotoxic conditions. We examined K1 knockdown to confirm anti-apoptotic effect of K1. Although non-specific siRNA (siRNA con) treated AK1 cells induced an increased level of DNA damage, K1 gene knockdown resulted in very little DNA damage (Fig. 2D). Under genotoxic stress, increased apoptotic cells in K1 knocked down AK1 were demonstrated by both TUNEL (Fig. 2E) and flowcytometry analyses (Fig. 2F). These results indicated that the expression of AIDS-related K1 induces resistance to apoptosis under genotoxic stress.

### AIDS-related K1 shows a derangement of the DNA damage response

It is widely accepted that viral gene expression induces DNA damage, which may lead to the suppression of cell cycle and cell senescence by DNA damage response (DDR) genes, such as ATM and Chk2^23–25^. Under these conditions, DDR associated proteins are activated by DNA damage, which induces a p53-dependent cell cycle arrest, apoptosis, and/or senescence^26,27^. However, it is believed that viral oncoproteins suppress and overcome DDR in the process of cellular transformation^24,28^. Because the present study showed that AK1 cells were highly proliferative and not apoptotic despite increased DNA damage, we speculated that AIDS-related K1 may suppress DDR, whereas classic K1 would have a smaller impact on DDR suppression. To investigate this issue, we analyzed the effect of K1 on the levels of DDR proteins with or without serum-free treatment to provoke genotoxic stress. Under non-genotoxic conditions, AK1 elicited a slight increase in Chk1 (Fig. 2G), suggesting that K1 gene expression cause a mild DNA damage response. Under genotoxic stress, all three cell lines showed elevated expressions of ATM, ATR, Chk1, and Chk2. However, AK1 cells had reduced levels of these proteins compared with mock cells (Fig. 2G). In contrast, CK1 cells exhibited comparable to or slightly decreased levels of these proteins relative to mock cells. These results suggest that DDR induction was impaired in AK1 cells, making the transformation activity of AIDS-related K1 more potent than that of classic K1.

### AIDS-related K1 induces both Akt and NF-κB activations through ITAM signaling activity

**Figure 3:**
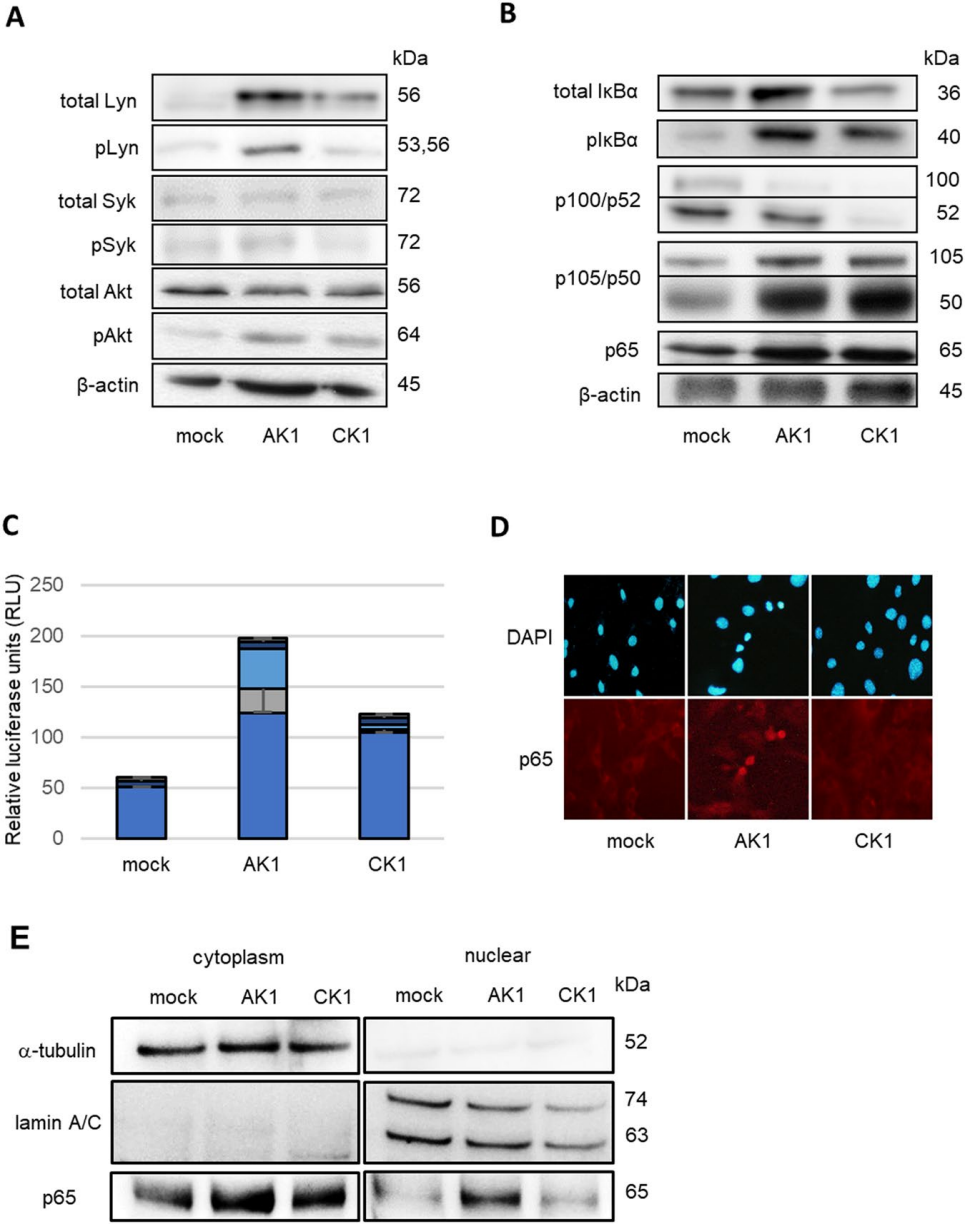
Comparison of ITAM activity and downstream signal pathway activity between AIDS-related K1 and classic K1. (**A**) AK1 cells induced significantly increased levels of total Lyn and phosphorylated Lyn and a slight increase of phosphorylated Syk, whereas CK1 cells exhibited weak expression of total Lyn. AK1 cells induced more potent phosphorylated Akt than CK1 cells. (**B**) AK1 cells induced marked phosphorylation of IκBα and p100 processing compared with CK1 cells. p105 processing of AK1 cells was identical to that of CK1 cells. (**C**) Luciferase assays showed the highest NF-κB activity in AK1 cells and moderate activity in CK1 cells. (**D**) Immunocytochemistry showed that nuclear translocation of p65 was observed in AK1 cells, but not in CK1 cells. Magnification, 400×. (**E**) p65 protein level in nuclear fraction of AK1 was higher than that of CK1.

K1 transduces intracellular signaling by activating Src family kinases, such as Syk and Lyn, the ITAM located in the cytoplasmic domain^11–13^. We next compared ITAM signaling activity between AK1 and CK1 cells. AK1 cells demonstrated augmented levels of Lyn and phosphorylated Lyn, and slightly increased phosphorylated Syk, compared with mock cells. In contrast, CK1 cells showed slightly increased levels of these molecules (Fig. 3A). To assess the downstream activity of AIDS-related K1 and classic K1, we compared the levels of Akt and NF-κB activities between AK1 and CK1 cells. AK1 cells demonstrated higher levels of phosphorylated Akt than CK1 cells (Fig. 3A). Also, luciferase assays showed higher NF-κB activity in AK1 cells than that in CK1 cells (Fig. 3C). Additionally, AK1 cells showed increased levels of phosphorylated IκBα, and p100 and p105 processing (Fig. 3B), as well as p65 nuclear translocation (Fig. 3D,E), demonstrating marked activation of both canonical and non-canonical NF-κB pathways. Although CK1 cells exhibited p105 processing comparable to that of AK1 cells, the levels of phosphorylated IκBα and p100 processing were weaker (Fig. 3B), indicating less NF-κB activity than AIDS-related K1.

### K1 gene knockdown in AK1 cells results in loss of transformation properties as well as decreased NF-κB and Akt pathways

**Figure 4:**
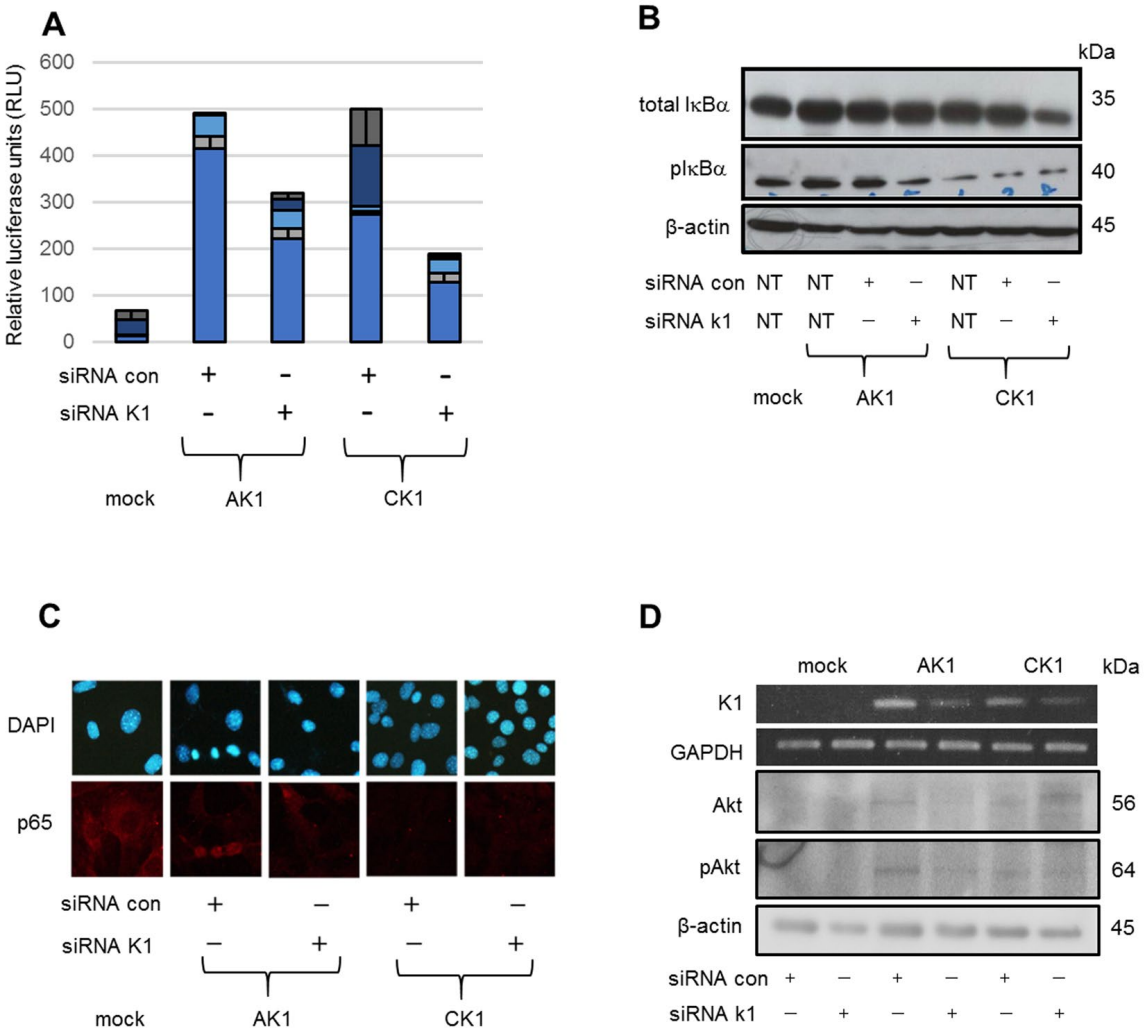
Effects of K1 gene knockdown on NF-κB and Akt signaling. (**A**) Luciferase assays showed that knockdown of the K1 gene resulted in decreased NF-κB activity in both AK1 and CK1 cells. (**B**) Knockdown of the K1 gene in AK1 cells demonstrated decreased levels of phosphorylated IκBα. (**C**) p65 was located in the nucleus of siRNA con-treated AK1 cells; however, K1 gene knockdown in AK1 cells exhibited a cytoplasmic localization of p65. Magnification, 400×. (**D**) K1 gene knockdown in AK1 cells induced a decreased level of phosphorylated Akt.

We next examined the effect of K1 knockdown on NF-κB and Akt pathways to assess the relationship between these signaling pathways and the transformation activity of K1. Luciferase assays showed that siRNA knockdown of K1 in AK1 and CK1 cells reduced NF-κB activity to nearly half the levels observed in each cell line treated with siRNA con (Fig. 4A). Moreover, siRNA knockdown of K1 in AK1 cells demonstrated decreased levels of phosphorylated IκBα (Fig. 4B). siRNA con-treated AK1 cells showed a nuclear p65 localization; however, knockdown of the K1 gene led to a cytoplasmic p65 localization shifted from the nucleus (Fig. 4C). Furthermore, siRNA knockdown of K1 in AK1 cells downregulated phosphorylated Akt. These results suggested that cellular transformation induced by K1 expression is associated with both NF-κB and Akt activities.

### Both AIDS-related and classic K1 show anchorage–independent growth

**Figure 5:**
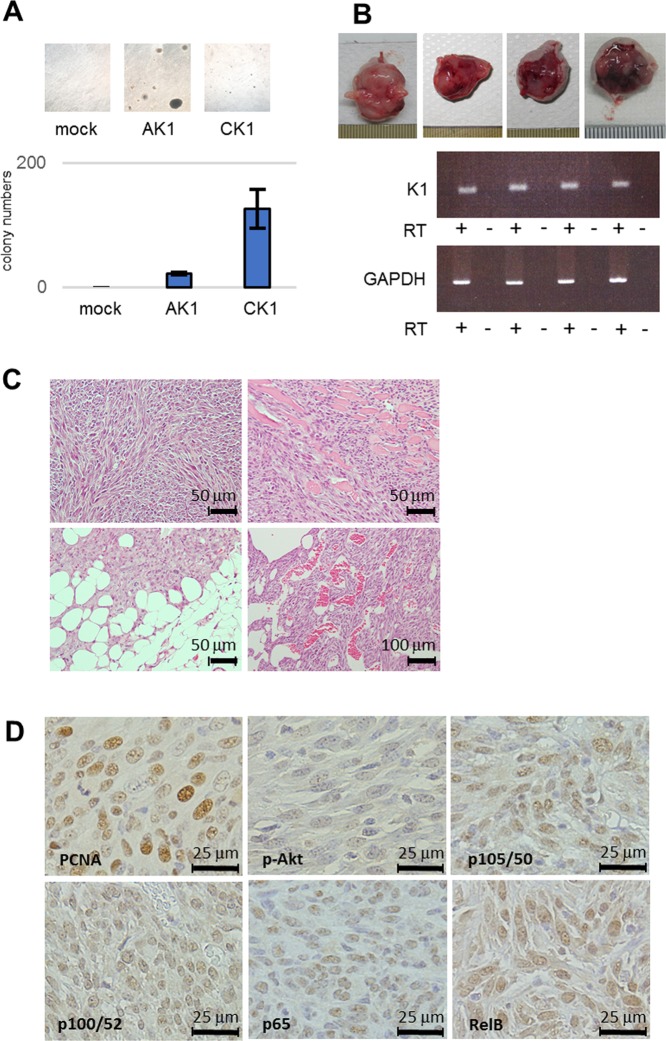
Anchorage-independent growth, and tumorigenicity in nude mouse of K1-expressing cells. (**A**) Colony formation assays demonstrated a small number of large colonies in AK1 cells and many small colonies in CK1 cells. Magnification, 100×. (**B**) Tumor formation in nude mice was observed in AK1 but not in CK1 or mock -injected nude mice. Large, lobulated tumors from the injection site on the back of the mice were removed from mice 61 days after AK1 injection. K1 expression in tumors from AK1-injected mouse was confirmed by RT-PCR. (**C**) Microscopic examination of the tumors revealed high grade sarcomatous histology with high mitotic figures (upper left) and invasion into the surrounding tissue (upper right and lower left). Primitive vascular formation was seen in the periphery of the tumors (lower right). Magnification, 400× (upper left, upper right and lower left) and 200× (lower right). (**D**) The tumor cells showed high PCNA positivity (68.2%). Although the tumor cells demonstrated low Akt activity, they induced high NF-κB signaling both canonical and non-canonical pathways. Magnification, 400×.

Because transformed cells show anchorage-independent growth, AK1 and CK1 cells were examined by the soft agar colony formation assay. Both AK1 and CK1 cells formed colonies during the 4-week observation period (Fig. 5A). On average, CK1 cells formed 120 colonies per 10^4^ plated cells, while AK1 cells formed 30 colonies; however, the colony size of AK1 cells was larger than that of CK1 (Fig. 5A). These data suggest that both AIDS-related K1 and classic K1 induced anchorage-independent growth, with the ability of anchorage-independent growth of AIDS-related K1 being more potent than that of classic K1.

### AIDS-related K1-expression induces tumor formation in nude mice, whereas classic K1 shows no tumor formation

Since AK1 cells showed increased proliferation rates, the resistance to apoptosis, derangement of DDR, and anchorage-independent growth, we speculate that these findings *in vitro* would reflect certain characteristics of transformation *in vivo*. To confirm whether the K1-expressing cells had acquired the full repertoire of neoplastic properties, we performed *in vivo* tumorigenicity experiments using nude mice. Cells (1 × 10^6^) of each cell line were injected into 5-week-old nude mice. After 12 weeks, no tumor was demonstrated in mice injected with either CK1 or mock cells (Table 1). In contrast, all AK1 cell-injected mice developed tumors at the injection site within 8 weeks. The tumors were irregular in shapes and sizes (10–20 mm in diameter), and contained hemorrhagic and/or necrotic centers (Fig. 5B). The borders between the tumors and adjacent normal tissues were macroscopically and microscopically inconspicuous, suggesting that the tumor cells were infiltrating the subcutaneous tissue. Histologically, the tumor cells were polygonal with enlarged nuclei and were arranged irregularly in the myxomatous stroma with a sarcomatous appearance (Fig. 5C upper left). Tumor cells at the periphery of the lesions were highly infiltrative, and many mitotic figures and high PCNA positivity (68.2%) were observed, suggesting a high proliferation index (Fig. 5C upper right and lower left and 5D). Primitive vascular-like structures were also observed at the peripheral regions of the tumors (Fig. 5C lower right). RT-PCR analysis identified K1 mRNA expression in the tumors of the nude mice, indicating that the expression remained stable (Fig. 5B). These results revealed that AIDS-related K1 has significant transformation activity compared with classic K1.

**Table 1:** Tumor formation in nude mice injected with MEFs expressing the KSHV K1 gene.

	Tumor formation in nude mice
mock	0/12
AK1	12/12
CK1	0/12

### The tumor formed by AIDS-related K1 activate NF-κB signaling

Because *in vitro* analysis of AK1 showed increased Akt and NF-κB activities, we examined *in vivo* Akt and NF-κB activities of the tumor caused by AK1 injection into nude mouse. Although phosphorylated Akt was only seen in small fraction (3.5%) of tumor cells, nuclear translocation of NF-κB such as p105/50, p100/52, p65 and RelB was observed, and the nuclear positive percentages were 70.6%, 82.2%, 84.4% and 82.5%, respectively (Fig. 5D). These results suggest that transformation activity of AIDS-related K1 is associated with increased NF-κB signaling both canonical and non-canonical pathways.

## Discussion

The current study demonstrated that more potent transformation activity of AIDS-related K1, with higher ITAM and the downstream pathway activities than those of classic K1. Furthermore, knockdown of K1 induced a loss of transformation activity and reduced downstream signaling. We revealed that the increased transformation activity of AIDS-related K1 is associated with the clinical aggressiveness of AIDS-related KS. In contrast, the weaker transformation activity of classic K1 appears to be correlated with a mild clinical presentation.

KSHV K1 is a transmembrane protein containing ITAM in the cytoplasmic region and is involved in transformation. When activated (i.e., with phosphorylated tyrosine residues), ITAM recruits specific Src kinases, such as Lyn and Syk. Subsequently, phosphorylated Lyn and Syk transduce downstream signaling by activating PI3K/Akt, mTOR, NF-κB, Wnt and MAPK pathways. In the present study, AIDS-related K1 induced higher Lyn activity compared with classic K1. Also, NF-κB and PI3K/Akt activities evoked by AIDS-related K1 were more potent than those of classic K1, which are each correlated with ITAM activity. K1 has been shown to induce transformation in rodent fibroblasts *in vitro* and it induces lymphoma in common marmosets *in vivo*^14^. K1 is also capable of immortalizing human umbilical vein endothelial cells (HUVECs) through the increased activity of the PI3K/Akt pathway^16^. Increased Lyn kinase activity in K1 transgenic nude mouse elicits lymphomagenesis by concomitant VEGF induction and increased NF-κB activity^13^. We observed tumor formation in AK1-injected nude mice, whereas no tumor formation was observed in CK1-injected mice. These findings indicate that the marked transformation activity of AIDS-related K1 is associated with higher ITAM signaling than classic K1. The fact that K1 knockdown in AK1 cells lost transformation activity also supports this hypothesis.

Besides the K1 gene, the KSHV genome encodes more than 80 genes, including many viral oncogenes such as LANA, viral D-type cyclin, viral FLICE-inhibitory protein, viral IL-6, viral CC chemokine homologs and viral G protein-coupled receptors^29^. The amino acid sequences of these viral oncogenes, with the exception of K1 and K15, are well conserved, whereas the amino acid sequences of K1 and K15 show great variability. Because of its vast degree of sequence variation, K1 is specifically used for KSHV classification by differentiating the genotypes A, B, C, and D^7,8^. Taking the correlation between sequence variability and the transformation activity of K1 into consideration, we assumed that the difference in virulence among the KSHV genotypes is derived from the transformation activity differences among the K1 subtypes. Mancuso *et al*. reported the association between the genotyping of KSHV for classic KS and its clinical presentation. The study revealed that the majority of rapid progressor is associated with genotype A, whereas slow progressor is associated with genotype C^30^, implicating the correlation between the KSHV genotype and clinical presentation. The KSHV genotypes A and C predominate in Europe, the United States, the Middle East, and Asia^7^. Intriguingly, genotype A is prevalent among AIDS-related KS patients, whereas genotype C is found not only in AIDS-related KS, but also classic, endemic, and iatrogenic KS^31–34^. In our previous study, sequence analysis revealed that AIDS-related K1 corresponds to genotype A, whereas classic type K1 corresponds to genotype C. Based on the findings of previous studies and our data, the differences in clinical presentation between AIDS-related KS and classic KS are associated with the transformation activity differences between AIDS-related K1 and classic K1.

Since AIDS-related KS arises as an opportunistic infection in AIDS patients and classic KS typically emerges in elderly people, KS tumorigenesis is likely associated with host immunity. Therefore, further studies employing K1-expressing cells under conditions simulating compromised immunity are needed to clarify the pathogenesis and differences in the clinical presentations of KS.

In conclusion, the transformation activity of AIDS-related K1 is more potent than that of classic K1 through its higher ITAM signaling, which leads to increased activity of the NF-κB and PI3K/Akt pathways. Our findings indicate that the differences in clinical presentations between AIDS-related KS and classic KS are associated with the differences in transformation activity between AIDS-related K1 and classic K1.

## Materials and Methods

### Cell culture

CF-1 primary MEFs and HeLa were purchased from American Type Culture Collection (Manassas, VA) and cultured in Dulbecco’s modified Eagle’s medium (DMEM) and Eagle’s minimum essential medium supplemented with 15% fetal bovine serum (FBS), respectively. TY-1, the KSHV-infected pleural effusion lymphoma cell line, was kindly provided by Dr. Katano (National Institute of Infectious Diseases, Japan) and cultured in RPMI1640 with 10% FBS. All cell lines were cultured at 37 °C with a humidity of 5% CO_2_.

### Plasmid constructs

The AIDS-related K1 and classic K1 genes were amplified using genomic DNA from AIDS-related KS and classic KS, respectively, by PCR using primers (shown in Supplementary Table S1) containing specific restriction enzyme recognition sites. The PCR products were digested with restriction enzymes and subcloned into the retrovirus vector plasmid pMSCV-neo (Clontech, Mountain View, CA) to yield pMSCV-neo-AIDS related K1 and pMSCV-neo-classic K1. The NF-κB-luc (Stratagene, La Jolla, CA) reporter plasmid and pGL4.74 (Promega, Madison, WI) were used for luciferase assays.

### Retrovirus production and construction of K1 gene-expressing MEFs

The procedure for retrovirus production and the construction of viral gene-expressing MEFs have been described previously^22^. Briefly, one day before transfection, the packaging cell line, PT-67 (Clontech), was plated on 60- mm plates and grown to 80% confluence. The plasmids pMSCV-neo- AIDS-related K1 and pMSCV-neo-classic K1 (10 μg each) were transfected into PT-67 cells using Lipofectamine 2000 (Invitrogen, Carlsbad, CA). The culture media were then collected at 24, 48, and 72 h after transfection and filtered through a 0.45-μm cellulose acetate membrane. The viral titers of the supernatants were determined by serial dilution ranged between 4 × 10^4^ and 1 × 10^6^ cfu/mL. MEFs were infected for 24 h with the MSCV retrovirus encoding AIDS-related K1 or classic K1 gene and placed under drug selection (neomycin) to obtain CF-1 AIDS-related K1 (AK1) and CF-1classic K1 (CK1). Viral gene expression in retrovirus-infected cells was confirmed by reverse transcription PCR (Supplementary Fig. S1A). As a negative control, CF-1 cells were infected with an empty retrovirus to yield CF-1 neo (mock).

### Cell proliferation assays

AK1, CK1 or mock cells were plated on 96-well plates at 1000 cells/well. Cell proliferation assays were performed every 24 h using the Cell Counting Kit-8 (CCK-8: Dojindo, Kumamoto, Japan), which utilizes [2-(2-methoxy-4-nitrophenyl)-3-(4-nitrophenyl)-5-(2,4-disulfophenyl)-2H-tetrazolium, monosodium salt], and is a modification of the MTT colorimetric assay.

### RT-PCR

Total RNA was extracted using RNeasy (Qiagen, Valencia, CA) and treated with recombinant DNase I (Takara, Shiga, Japan) at 37 °C for 30 min. After adding EDTA and incubating at 80 °C to inactivate DNase, cDNA synthesis was performed using SuperScript III (Invitrogen) at 42 °C for 50 min. cDNA synthesis was confirmed by the PCR amplification of glyceraldehyde-3-phosphate dehydrogenase (GAPDH).

### Real-time PCR

To compare the gene expression among the clones, cDNA was treated with Brilliant II SYBR Green QPCR Master Mix (Agilent Technologies, Santa Clara, CA) and the reagent was analyzed by Mx3000P (Agilent Technologies) and StepOnePlus (Thermo Fisher Scientifics) using GAPDH as the internal control. The primer sequences for real time PCR were shown in Supplementary Table S1. Expression levels were calculated by the Comparative Ct method using the following formula:

$${\rm{The}}\,{\rm{expression}}\,{\rm{level}}\,{\rm{of}}\,{\rm{the}}\,{\rm{gene}}\,{\rm{of}}\,{\rm{interest}}={({\rm{1}}+{\rm{Eff}})}^{{\rm{Ct}}:{\rm{S}}-{\rm{Ct}}:{\rm{H}}}$$

$${\rm{Eff}}=[{10}^{(-1/{\rm{Slope}})}]-1,\,{\rm{Ct}}:{\rm{S}}={\rm{Ct}}\,{\rm{of}}\,{\rm{gene}}\,{\rm{of}}\,{\rm{interest}},\,{\rm{Ct}}:{\rm{H}}={\rm{Ct}}\,{\rm{of}}\,{\rm{GAPDH}}$$

### Immunocytochemistry

AK1 and CK1 cells were fixed in 4% paraformaldehyde (PFA) for 15 min, followed by washing with phosphate-buffered saline (PBS). Fixed cells were permeabilized with 0.25% Triton X-100 for 3 min, washed with PBS, and treated with 0.3% hydrogen peroxide to inactivate endogenous peroxidase. After blocking with 10% bovine serum albumin (BSA) for 2 h, cells were incubated at room temperature for 1 h with primary antibodies that recognize proliferating cell nuclear antigen (PCNA: Abcam, Cambridge, MA), γ-H2AX (Gene Tex, Irvine, CA), or p65 (Santa Cruz, Santa Cruz, CA). PCNA immunostaining was visualized using an LSAB kit (DAKO, Glostrup, Denmark) with 3, 3′-diaminobenzidine (DAB) as the chromogen. The γ-H2AX and p65 signals were detected using Alexa Fluor 594 (Invitrogen) and observed using EclipseTE300 (Nikon, Tokyo, Japan) and Bz9000 (Keyence, Osaka, Japan) fluorescence microscopes.

### Cell cycle analysis by flow cytometry

A total of 10^5^ transfected K1 expressing MEFs were washed with PBS and fixed with ice-cold 100% ethanol for 2 h, washed again with PBS, and stained with DAPI for 30 min. The DNA content of the cells was analyzed using a flow cytometer (MAQSQuant Analyzer; Miltenyi Biotec, Bergisch Gladbach, Germany).

### Detection of apoptosis

Apoptotic cells were detected by TdT-mediated dUTP nick end labeling (TUNEL). The cells were plated on the chamber slides (Nunc, Roskilde, Denmark) at a concentration of 10^5^ cells/slide and cultured with or without genotoxic treatments such as serum-free medium or 250 μM hydrogen peroxide treatments. Cells were then fixed with 4% PFA for 15 min. After washing with PBS, the slides were treated with 0.3% hydrogen peroxide to block endogenous peroxidase. After washing with PBS, TUNEL staining was performed using the *in-situ* Apoptosis Detection kit (Takara) which label 3′-OH ends of fragmented DNA with fluorescein dUTP and visualized under an Eclipse TE300 fluorescence microscope. The degree of apoptosis was calculated by the percentage of TUNEL positive signals. For each cell line, at least 97 cells were evaluated, and three TUNEL experiments were analyzed. Only nuclear positive signals were used for analysis. Apoptotic cells were also detected by a flow cytometer (MACSquant) using Annexin V-fluorescein isothiocyanate (FITC) and propidium iodine (PI), which detect Annexin V-accessible internal phosphatidylserine and permeabilized cell membrane, respectively.

### Detection of reactive oxygen species (ROS)

ROS generation was analyzed by chloromethyl dichlorodihydrofluorescein diacetate (CM-DCF-DA, Molecular Probe, OR). One day before the cells were plated on 96-well Optical Btm Plt PolymerBase White/Lid Cell Culture Sterile PS (Thermo Fisher Scientific, Rochester, NY) at the concentration of 5000 cells/well. After washing with PBS, the cells were treated with 1 μM CM-DCF-DA for 15 min, and the fluorescence (excitation 485 nm, emission 538 nm) was measured spectrofluorometrically with a Fluoroskan Ascent^®^ FL system (Thermo Fisher Scientific, Vantaa, Finland). The cells were also observed under an Eclipse TE300 fluorescence microscope.

### Western blot analysis

Total cell protein extracts were obtained using RIPA lysis buffer supplemented with a cocktail of protease inhibitors (Roche, Manheim, Germany). Protein concentration was measured using the Bradford protein assay (Bio-Rad, Hercules, CA). Protein samples (25 μg) were separated by electrophoresis on 12% sodium dodecyl sulfate-polyacrylamide gels. Separated proteins were then transferred to polyvinylidene difluoride membranes (Millipore Immobilon-P, Merck, Burlington, MA). Protein-bound membranes were blocked in 5% nonfat milk solution (0.1% Tween 20 in PBS) and incubated in one of the following primary antibodies: anti-Akt, anti-phosphorylated Akt, anti-phosphorylated Syk, anti-IκBα, anti-phosphorylated IκBα, anti-p21, anti-p100/p52 and anti-p105/p50 (Abcam), anti-Lyn, anti-phosphorylated Lyn, anti-Syk, anti-p65, anti-α tubulin, and anti-β actin (Cell Signaling Technology, Danvers, MA), and anti-lamin A/C (Upstate, Lake Placid, NY). After washing with PBS three times, antibody reactions were analyzed using Amersham ECL plus Western blotting detection reagents (GE Healthcare, Buckinghamshire, UK). Nuclear and cytoplasmic fractions were separated by NE-PER nuclear and cytoplasmic extraction reagents (Thermo Fisher Scientific, Rockford, IL) according to the manufacturer’s instruction. The protein expression level was quantified by densitometric analysis using ImageJ (National Institute of Health, Bethesda, MD, http://imagej.nih.gov/ij/)^35^. Relative protein expression of gene of interest was calculated using β actin signal as an internal control.

### Small interfering RNA (siRNA) transfection

For K1 knockdown, siRNAs were designed and synthesized (Invitrogen). The sequences of the siRNA duplexes for K1 and non-specific siRNA controls are shown in the Supplementary Table S1. One day prior to siRNA transfection, MEFs transduced with one kind of MSCV retrovirus were plated at 5 × 10^4^ cells/well on 6-well plates. K1 siRNA or control (non-specific) siRNA were then transfected into the cells using Lipofectamine 2000 according to the manufacturer’s instructions and analyzed 48 h after transfection. The specificities of the siRNAs for K1 are shown in Supplementary Fig. S1B.

### Luciferase assay

K1 expressing cells were transfected with the reporter plasmid NF-κB-luc, control plasmid pGL4.74 and siRNA. The total amount of transfected DNA and siRNA were equalized by the addition of empty vectors or non-specific siRNAs, respectively. After 48 h, cell lysates were assayed for firefly luciferase activity using the dual luciferase assay system (Promega, Madison, WI). Relative activities were calculated using Renilla luciferase activity as an internal control for transfection.

### Soft agar colony formation assay

Base agar (0.5% agar, 1× DMEM/F12, and 10% FBS) was added to 6-well plates and allowed to solidify, followed by top agar (0.35% agarose, 1× DMEM/F12, and 10% FBS) containing 1 × 10^4^ trypsinized cells. After the top agar solidified, a small amount of 1 × DMEM/F12 with 10% FBS was added to each well. Cells were then incubated at 37 °C in a humidified incubator. The culture medium was changed every 3 days. Colony formation was assessed by observing the cells under an inverted microscope for 4 weeks.

### Animal experiments

Animal experiments were performed in accordance with the guidelines for animal treatment, housing, and euthanasia of the Animal Experiment Committee of the University of the Ryukyus. The experimental protocol was approved by the Animal Experiment Committee of the University of the Ryukyus (The number of Approval: 6070). Four-week-old female BALB/cScl-nu/nu nude mice were purchased from Japan SLC (Shizuoka, Japan). At 5–7 weeks of age, mice were injected subcutaneously in the back with 2 × 10^6^ retrovirally-infected MEFs. After 12 weeks, the mice were euthanized by intra-abdominal injection of sodium pentobarbital, and tissue samples were collected for molecular and histological analyses.

### Histological examination

Tumors from the nude mice were fixed in 10% phosphate-buffered formalin, embedded in paraffin, and sectioned into 3 μm thick sections. Sections were then dewaxed with xylene and rehydrated in a graded ethanol series, processed, and stained with hematoxylin and eosin. Immunohistochemistry was performed using one of the following primary antibodies: anti-PCNA, anti-phosphorylated Akt, anti-p105/50, anti-p100/52, anti-p65 and anti-RelB (Cell Signaling Technology). The details of the staining methods have been described in a previous report^36^.

### Statistical analysis

In cell proliferation assay, Non-repeated measures ANOVA was used for evaluating the difference among groups. When significant differences were found, the Turkey test was performed. Mann-Whitney U-test was used for evaluating the difference between two groups. Other data were analyzed by the Kruskal-Wallis H test. When significant differences were found, the Mann-Whitney test with Bonferroni correction was carried out to evaluate the differences between each group. P values of less than 0.05 were considered statistically significant.

## Supplementary information

Supplementary information

## Data Availability

The datasets used and/or analyzed during the current study are available from the corresponding author on reasonable request.
