# Supplementary material for: K1 gene transformation activities in AIDS-related and classic type Kaposi’s sarcoma: Correlation with clinical presentation
Source: Sci Rep. 2019 Apr 23;9:6416. doi: 10.1038/s41598-019-42763-0 (PMC6478685; doi:10.1038/s41598-019-42763-0)
Supplement: Supplementary file 1 — Supplementary information [file 41598_2019_42763_MOESM1_ESM.pdf]

Supplementary information

K1 gene transformation activities in AIDS-related and classic type Kaposi's sarcoma: Correlation with clinical presentation

Ayumi Tamanaha-Nakasone [1#], Karina Uehara [1,2#], Yasuka Tanabe [1], Haruna Ishikawa [1], Natsuko Yamakawa [1], Zensei Toyoda [1], Kiyoto Kurima [1], Shinichiro Kina [2,3], Masayuki Tsuneki [4], Yuko Okubo [5], Sayaka Yamaguchi [5], Daisuke Utsumi [5], Kenzo Takahashi [5], Hirofumi Arakawa [6], Akira Arasaki [2], Takao Kinjo [1*]

Figure S1

S1A

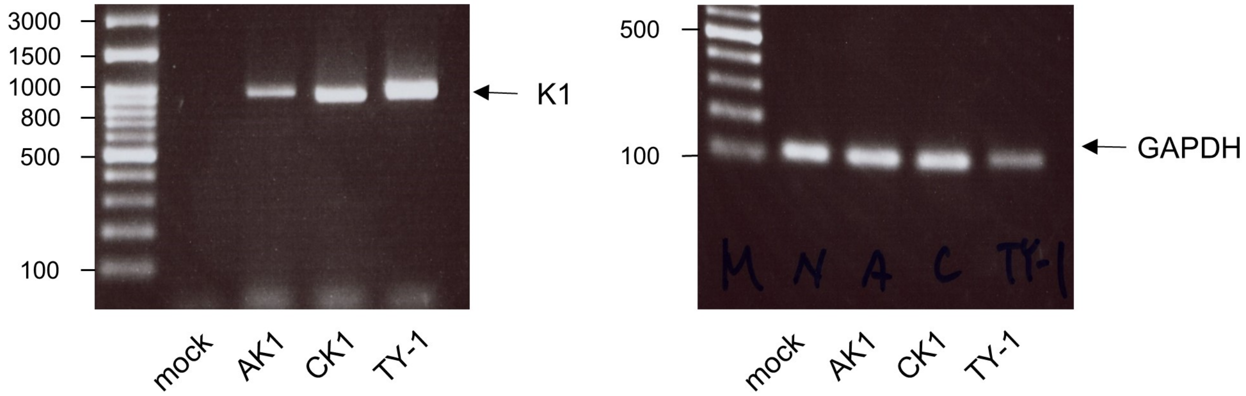

S1B

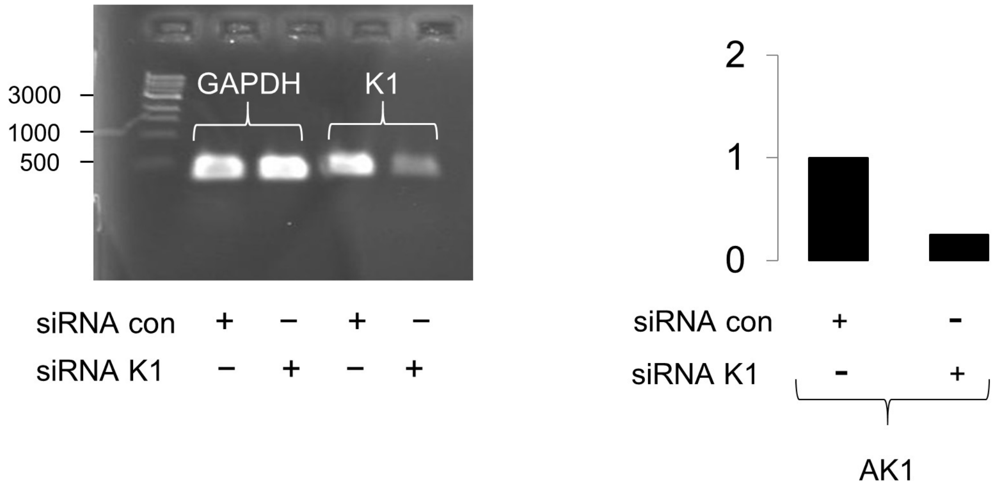

(S1A) RT-PCR analysis of MEFs infected with retrovirus demonstrated expression of AIDS-related K1 (AK1) and classic K1 (CK1). TY-1, the KSHV-infected pleural effusion lymphoma cell line, was used for positive control.

(S1B) AK1 cells were treated with either siRNA K1 or siRNA con. The specificity of knockdown of the K1 gene was confirmed by RT-PCR.

Figure S2: original blots shown in figures

Fig. 1D

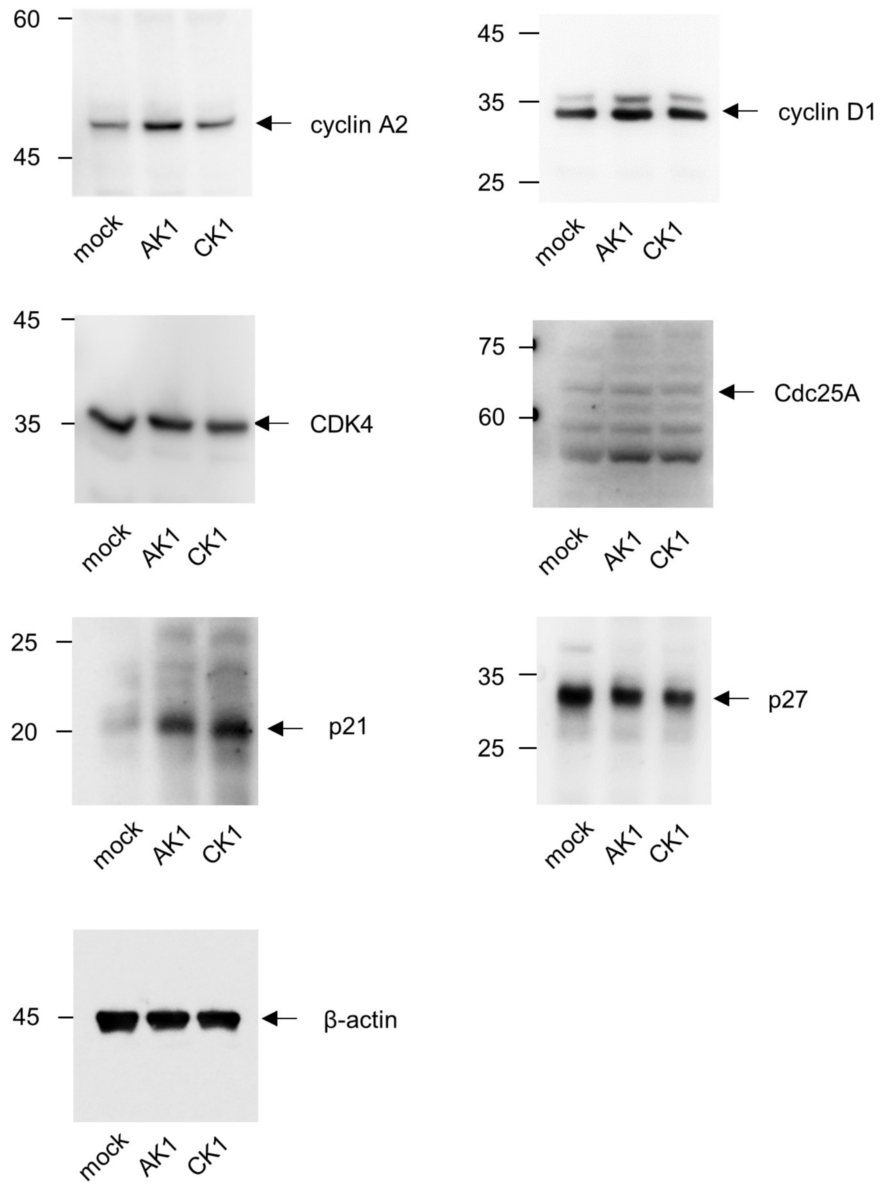

Fig. 2G

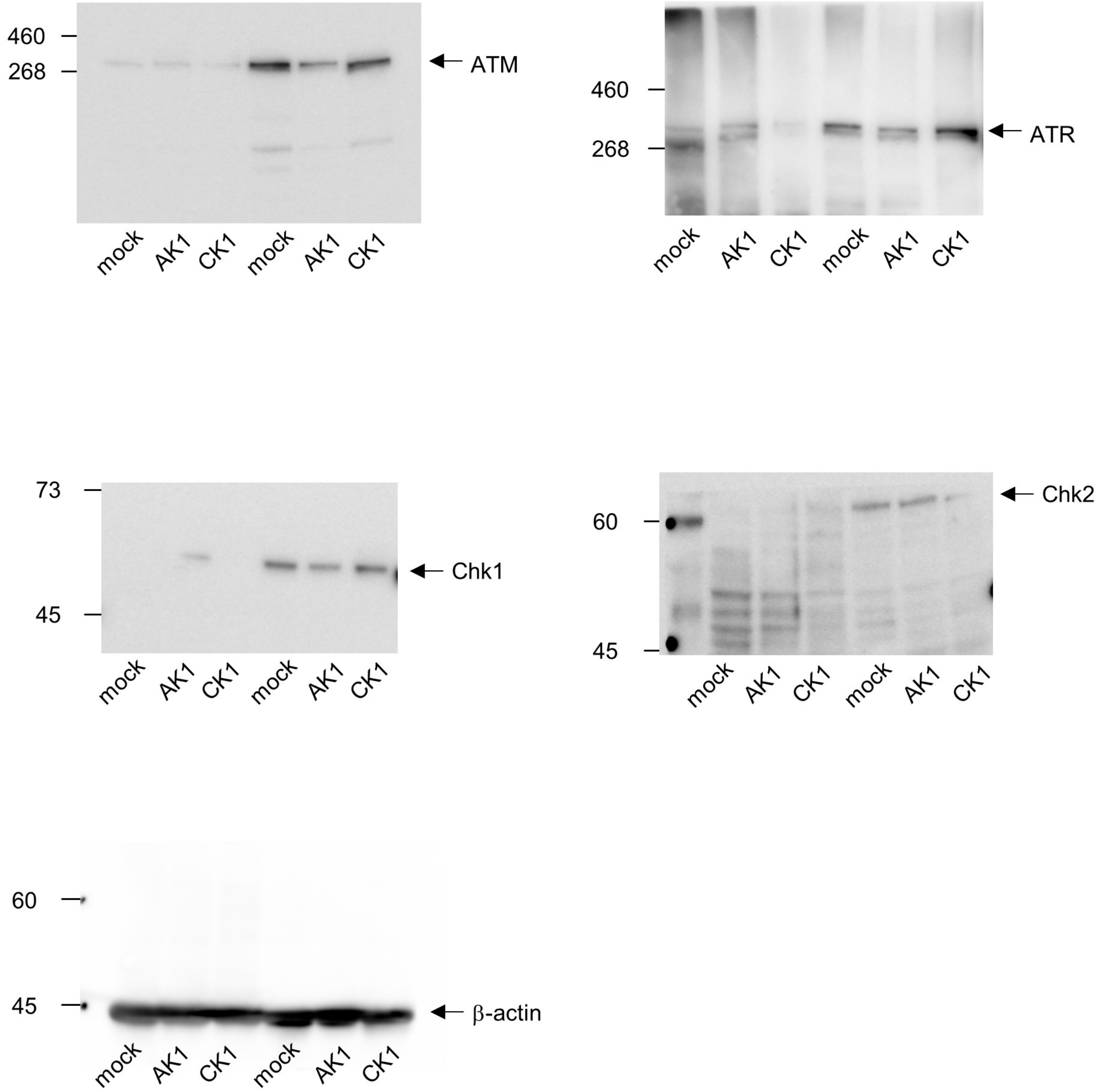
460
268
ATM
mock
AK1
CK1
mock
AK1
CK1
460
268
ATR
mock
AK1
CK1
mock
AK1
CK1
73
45
Chk1
mock
AK1
CK1
mock
AK1
CK1
60
45
Chk2
mock
AK1
CK1
mock
AK1
CK1
60
45
β-actin
mock
AK1
CK1
mock
AK1
CK1

Fig. 3A

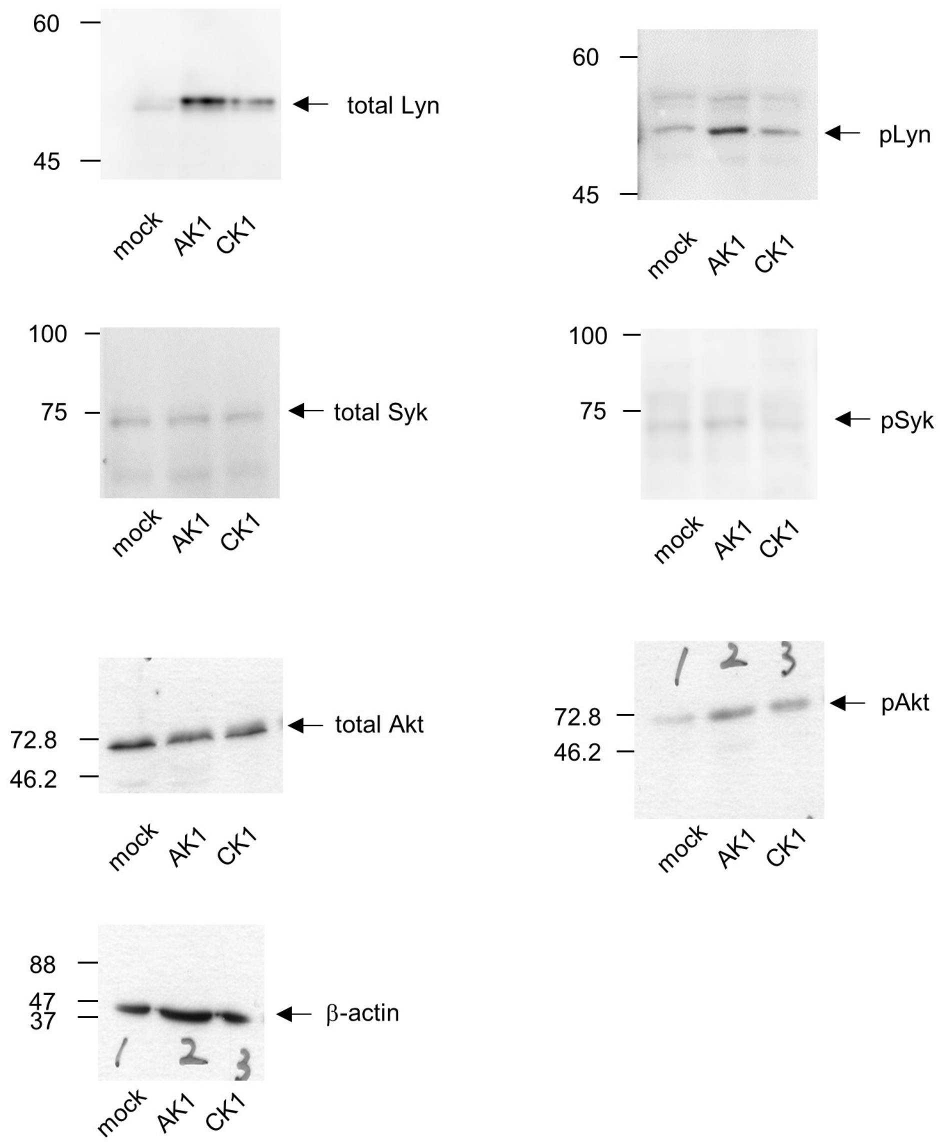
60
45
total Lyn
mock
AK1
CK1
60
45
pLyn
mock
AK1
CK1
100
75
total Syk
mock
AK1
CK1
100
75
pSyk
mock
AK1
CK1
72.8
46.2
total Akt
mock
AK1
CK1
1 2 3
72.8
46.2
pAkt
mock
AK1
CK1
88
47
37
β-actin
1 2 3
mock
AK1
CK1

Fig. 3B

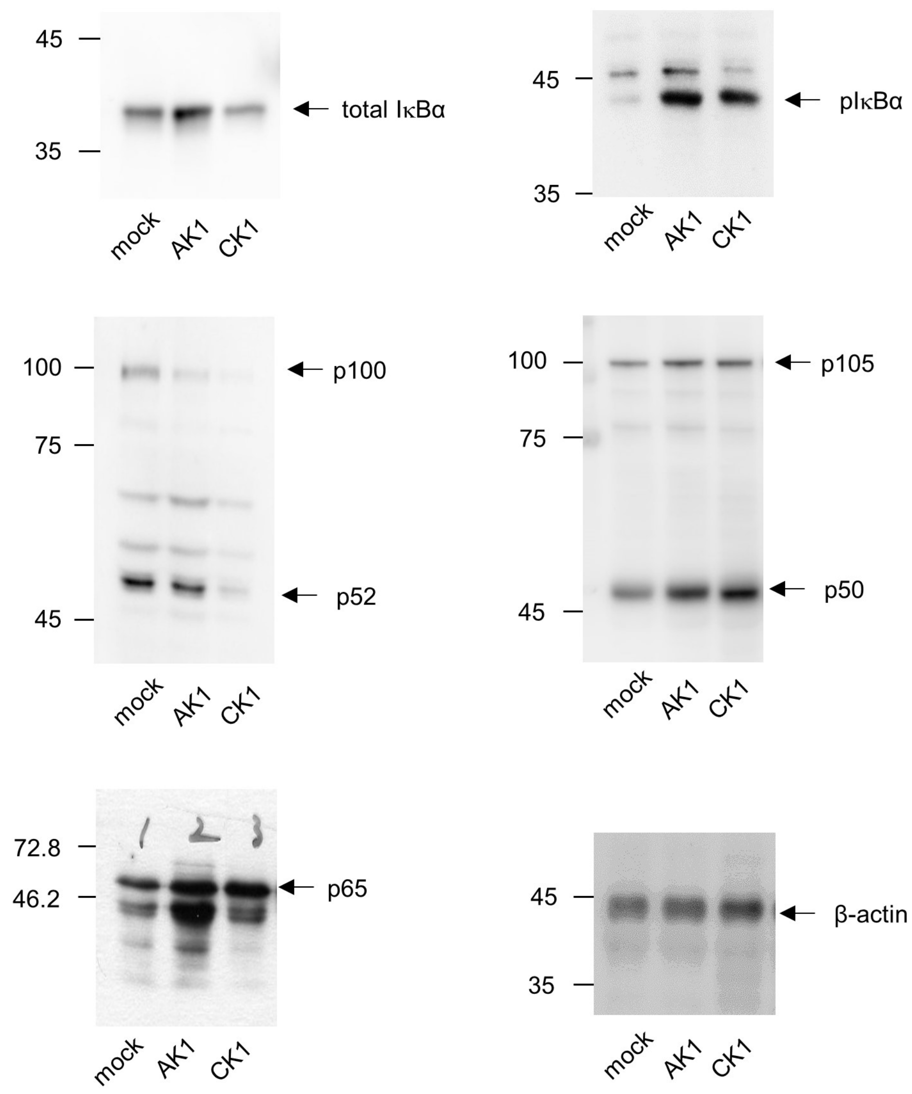
45
35
total IκBα
mock
AK1
CK1
45
35
pIκBα
mock
AK1
CK1
100
75
45
p100
p52
mock
AK1
CK1
100
75
45
p105
p50
mock
AK1
CK1
72.8
46.2
p65
mock
AK1
CK1
45
35
β-actin
mock
AK1
CK1

Fig. 3E

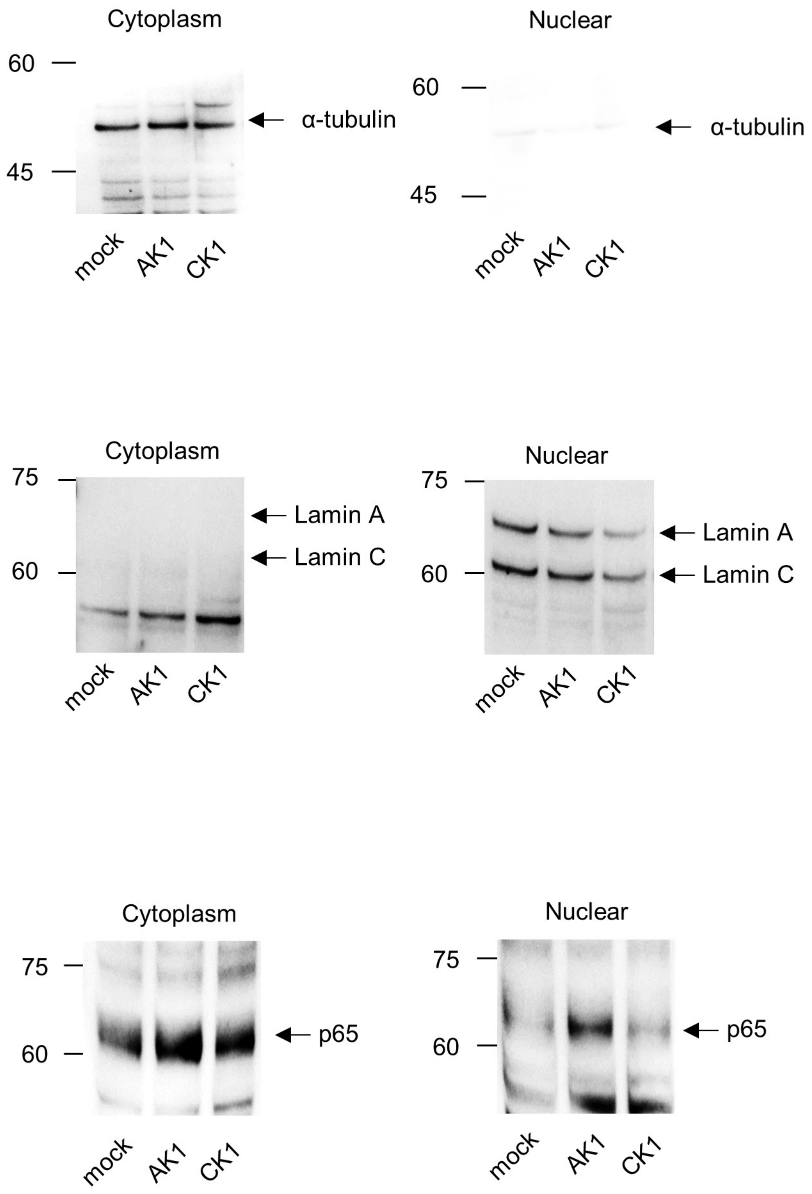

Cytoplasm
60
45
α-tubulin
mock
AK1
CK1
Nuclear
60
45
α-tubulin
mock
AK1
CK1
Cytoplasm
75
60
Lamin A
Lamin C
mock
AK1
CK1
Nuclear
75
60
Lamin A
Lamin C
mock
AK1
CK1
Cytoplasm
75
60
p65
mock
AK1
CK1
Nuclear
75
60
p65
mock
AK1
CK1

Fig. 4B

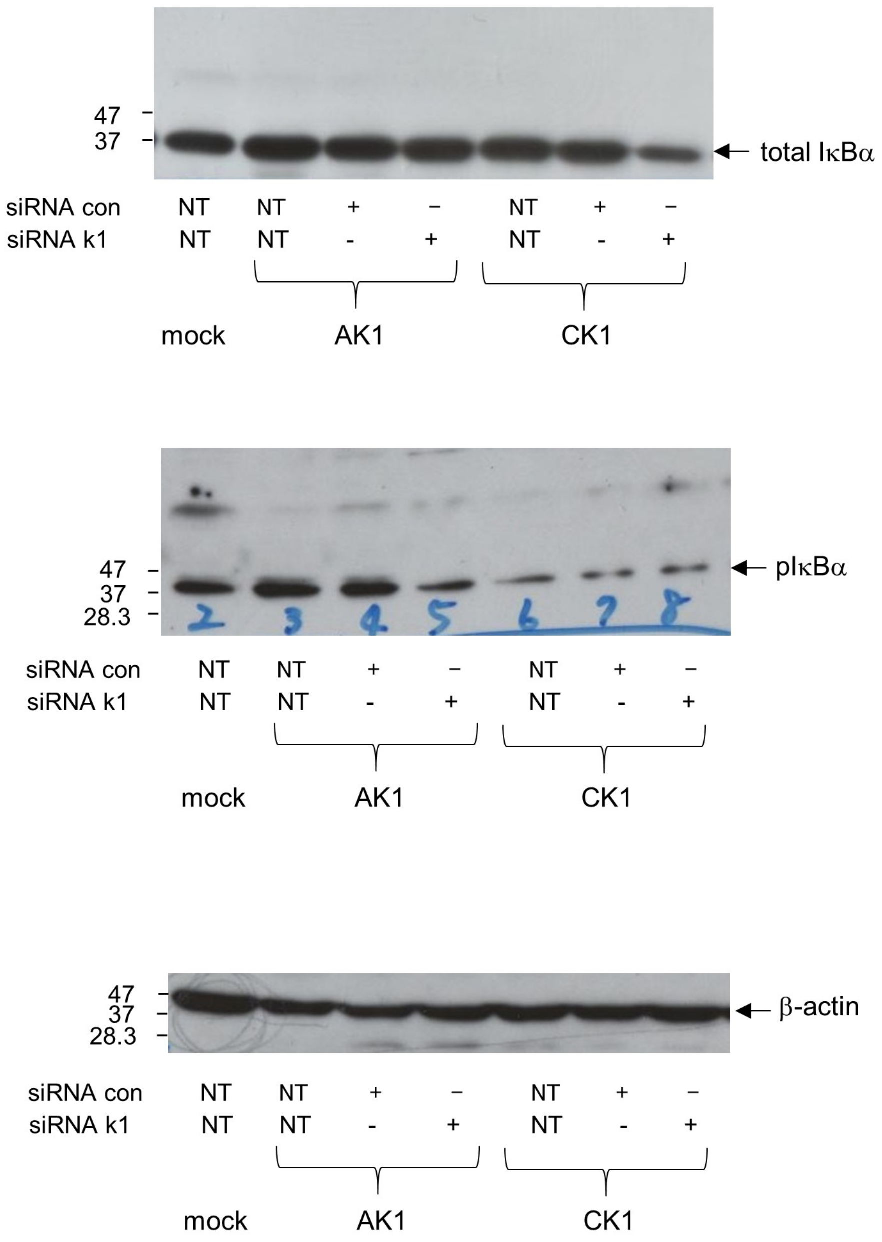
47
37
total IκBα
siRNA con NT NT + − NT + −
siRNA k1 NT NT - + NT - +
mock
AK1
CK1
47
37
28.3
pIκBα
2 3 4 5 6 7 8
siRNA con NT NT + − NT + −
siRNA k1 NT NT - + NT - +
mock
AK1
CK1
47
37
28.3
β-actin
siRNA con NT NT + − NT + −
siRNA k1 NT NT - + NT - +
mock
AK1
CK1

Fig. 4D

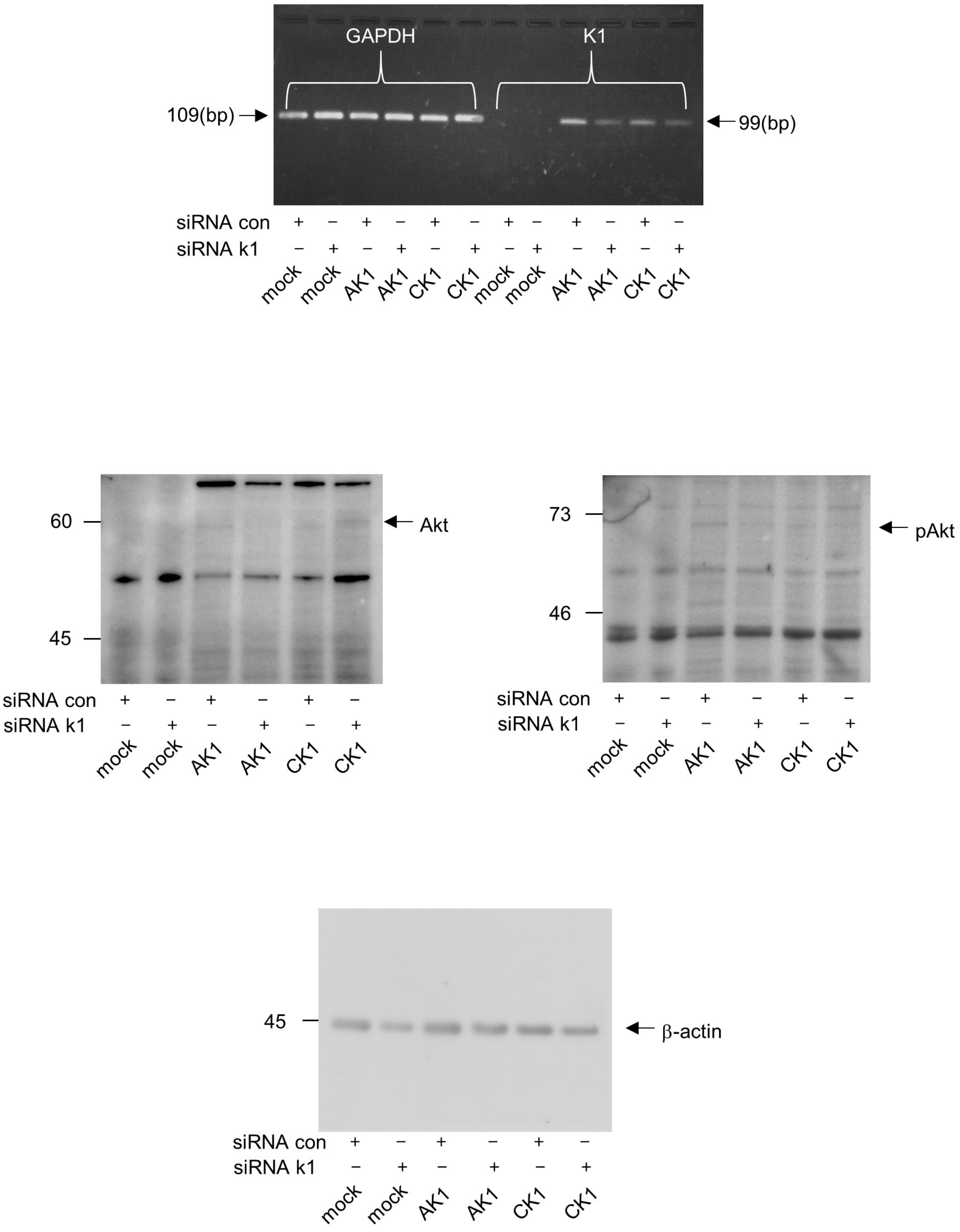
GAPDH
K1
109(bp)
99(bp)
siRNA con + − + − + − + − + − + −
siRNA k1 − + − + − + − + − + − +
mock mock AK1 AK1 CK1 CK1 mock mock AK1 AK1 CK1 CK1
60
45
Akt
siRNA con + − + − + −
siRNA k1 − + − + − +
mock mock AK1 AK1 CK1 CK1
73
46
pAkt
siRNA con + − + − + −
siRNA k1 − + − + − +
mock mock AK1 AK1 CK1 CK1
45
β-actin
siRNA con + − + − + −
siRNA k1 − + − + − +
mock mock AK1 AK1 CK1 CK1

Supplementary Table S1. The primers and siRNAs used in this study.

Name		sequence
K1	F	5'-ggaattctactaattttgaaaggcggg-3'
	R	5'-ccgctcgagggccatgctgtaagtagcac-3'
classic K1	F	5'-ggaattcgaccttgttggacatcccgta-3'
	R	5'-ccgctcgagtcagtaccaatccactggttgcgtatagtcttcc-3'
K1(199-216)	F	5'-tgatttcaacgccttacacgt-3'
K1(622-601)	R	5'-gccgaaaaacatagacgatttg-3'
K1 (RT)	F	5'-caaacggacgaaatgaaacc-3'
	R	5'-acgacagcccgttagaacaa-3'
GAPDH (RT)	F	5'-aaatggtgaaggtcggtgtg-3'
	R	5'-atgaaggggtcgttgatgg-3'
GAPDH	F	5'-atgacaactttggcattgtg-3'
	R	5'-gaagagtgggagttgctgtt-3'
cyclin A2	F	5'-tgggttcttctctggctccaa-3'
	R	5'-gcctggagatgggagcgtta-3'
cyclinD1	F	5'-ctgtcgctggagcccgtgaaaaag-3'
	R	5'-gaagttgttggggctcctcaggtt-3'
CDK4	F	5'-atggctgccactcgatatgaaccc-3'
	R	5'-gtaccagagcgtaaccaccacagg-3'
Cdc25a	F	5'-acccctactagcatggtaagcc-3'
	R	5'-tctcttcaacaccgcccgag-3'
p21	F	5'-cgcggtgtcagagtctaggg-3'
	R	5'-accgaagagacaacggcaca-3'
p27	F	5'-tctcttcggcccggtcaatc-3'
	R	5'-ctgccactcgtatctgccct-3'
siRNA-control		5'-gcccuaaagauggccagccaucuuu -3'
siRNA-K1		5'-auaauaauccugcaauuguuguggc -3'
